# Wild mushroom consumption susceptibility among Chinese university students: A machine learning study

**DOI:** 10.1371/journal.pone.0345659

**Published:** 2026-03-24

**Authors:** Yu Chen, Xinjie Zhao, Ying Yue, Zhenyi Li, Si Chen

**Affiliations:** 1 School of Art and Communication, Fujian Polytechnic Normal University, Fuqing, China; 2 School of Journalism and Communication, Peking University, Beijing, China; 3 China National Center for Food Safety Risk Assessment, Beijing, China; 4 Department of Communication and Culture, Royal Roads University, Victoria, BC, Canada; Federal University Otuoke, NIGERIA

## Abstract

**Objectives:**

To investigate factors associated with susceptibility to wild mushroom consumption using machine learning approaches and identify key predictors for targeted intervention development.

**Methods:**

A cross-sectional survey of 216 Chinese university students employed three machine learning algorithms (Logistic Regression, Random Forest, Extremely Randomized Trees [ExtraTrees]) to predict consumption susceptibility based on demographics, media usage, and cognitive factors. Susceptibility was assessed through scenario-based questions following established frameworks from tobacco research. Model performance was evaluated using AUC with 95% confidence intervals calculated via bootstrap resampling (1,000 iterations). Sensitivity analyses were conducted using alternative susceptibility thresholds.

**Results:**

65.3% were classified as susceptible to consumption. Logistic Regression achieved highest performance (AUC = 0.776, 95% CI: 0.679–0.862). Risk perception emerged as the strongest predictor (importance = 0.133 ± 0.044), followed by mushroom picking experience (0.101 ± 0.017) and content impression (0.089 ± 0.018). Among the 63 participants (29.2%) who reported using AI models, 75.93% indicated trust levels of ‘fairly trust’ or above.

**Conclusions:**

In this exploratory study of Chinese university students from a single institution, cognitive factors, particularly risk perception and identification ability, showed the strongest associations with consumption susceptibility. These preliminary findings suggest that targeted interventions enhancing risk awareness may be relevant for this population, though replication across diverse samples is needed before broader conclusions can be drawn.

## 1. Introduction

Wild mushroom poisoning constitutes a critical public health challenge, affecting tens of thousands annually worldwide [1,2]. The World Health Organization identifies it as a priority surveillance area, with mortality rates exceeding 90% for cyclopeptide-containing species like Amanita phalloides [3,4]. In 2024, China alone documented 599 incidents affecting 1,486 patients, representing a 19% increase from 2023 [5].

### 1.1. Prevention challenges and China’s response

The complexity of species identification, combined with cultural traditions of wild mushroom foraging, creates persistent challenges for prevention efforts in China and other regions with mushroom consumption traditions. Previous research has documented regulatory approaches in various countries, though the effectiveness of different strategies remains context-dependent [6].

China has implemented a comprehensive “Four Don’ts” prevention policy: (1) Don’t pick unfamiliar wild mushrooms, (2) Don’t buy wild mushrooms from informal vendors, (3) Don’t eat wild mushrooms without proper identification, and (4) Don’t share wild mushrooms with others without certainty of safety [7]. Despite these efforts, poisoning incidents persist, highlighting the need for understanding behavioral factors underlying consumption decisions.

### 1.2. Susceptibility as a predictive framework

Susceptibility, originally developed in tobacco research, represents an individual’s cognitive predisposition to engage in a risk behavior before actual experimentation occurs [8]. Pierce and colleagues (1996) defined susceptibility as “the absence of a firm decision not to smoke,” measured through intentions and expectations for future behavior. This preexperimentation phase precedes early experimentation, making it a crucial target for prevention interventions. The predictive validity of susceptibility has been demonstrated across multiple health behaviors, establishing it as a robust framework for understanding behavioral risk [8].

In the context of wild mushroom consumption research, susceptibility represents an individual’s cognitive predisposition to consume wild mushrooms, closely related to their knowledge level, attitudes, perceptions, and social environment. This study adapts the susceptibility framework to assess university students’ behavioral intentions toward wild mushroom consumption across three scenarios: recent consumption, future consumption within one year, and consumption when invited by friends.

### 1.3. Theoretical integration and study objectives

Understanding wild mushroom consumption behavior requires theoretical integration spanning health behavior science, communication theory, and cognitive psychology. The Health Belief Model (HBM) emphasizes perceived susceptibility, severity, benefits, and barriers in health decision-making [9,10]. The Theory of Planned Behavior (TPB) highlights attitudes, subjective norms, and perceived behavioral control influences on behavioral intentions [11]. Protection Motivation Theory (PMT) integrates threat appraisal with coping appraisal mechanisms [12].

The emergence of artificial intelligence (AI) models as information sources represents a relatively new phenomenon that may be relevant to health communication. Recent systematic reviews highlight AI’s growing role in health communication and education [13,14]. Understanding university student interactions with emerging technologies may be relevant for developing effective health communication strategies.

This exploratory research aims to: (1) identify factors associated with wild mushroom consumption susceptibility among Chinese university students using machine learning approaches; (2) evaluate relative importance of different predictor categories; and (3) describe patterns of AI model usage and trust in this population. Given the exploratory nature of this study and the convenience sample, findings should be considered hypothesis-generating rather than confirmatory.

## 2. Methods

### 2.1. Study design and participants

This cross-sectional study was conducted at a university in Fujian Province, China, between August and September 2024. Using convenience sampling, we initially recruited 246 university students. After excluding questionnaires with completion times under 90 seconds, 216 valid responses were obtained, yielding a response rate of 87.8%.

University students were selected as the study population because: (a) they represent an understudied yet potentially relevant population for wild mushroom consumption susceptibility research; (b) they are in a developmental period where health behaviors are being established; and (c) they have high exposure to social media content, including content that may normalize risky behaviors. However, this population may not represent the demographic groups at highest risk for mushroom poisoning, which is acknowledged as a limitation.

The sample size of 216 participants for 17 predictor variables yields a ratio of approximately 12.7:1, which meets the generally accepted minimum threshold of 10:1 for machine learning applications while acknowledging that larger ratios would enhance model stability.

### 2.2. Data collection instrument

The questionnaire was developed based on established theoretical frameworks and validated scales, culturally adapted for the Chinese context. The instrument comprised four main sections:

**Demographic and behavioral characteristics:** Age, gender, academic year, geographic origin, and behavioral factors including smoking, drinking, and social venue visitation patterns.

**Media Usage and Information Exposure:** Media consumption patterns across eight platforms (newspapers/magazines, television, radio, information websites, domestic social media, short video platforms, AI models, and international social media), trust evaluations using 7-point scales (1 = never used, 2 = completely distrust, 7 = completely trust), and exposure to wild mushroom-related content. Note: “AI models” refers to large language model-based conversational AI systems such as ChatGPT, Kimi, Doubao, and similar applications. The trust scale combines usage and trust assessment, with “1 = never used” representing non-users and values 2–7 representing trust levels among users. This combined approach is acknowledged as a limitation.

**Cognitive Assessment:** Knowledge evaluation across four dimensions: (1) identification ability (3 items), (2) prevention knowledge (3 items), (3) treatment knowledge (3 items), and (4) risk perception (4 items). Items were measured using 5-point Likert scales (1 = strongly disagree, 5 = strongly agree).

**Susceptibility Assessment:** Following established susceptibility measurement approaches [8], this study assessed susceptibility to wild mushroom consumption through three scenario-based questions: (1) “Have you tried eating wild mushrooms recently?”, (2) “Do you think you will try eating wild mushrooms in the next year?”, and (3) “If your best friend offered you wild mushrooms in the next year, would you eat them?”. Each scenario used 4-point scales (1 = very unlikely, 4 = very likely).

### 2.3. Variable construction and statistical analysis

**Outcome Variable:** Following established susceptibility classification protocols [8], participants were classified as susceptible to wild mushroom consumption if they indicated anything other than “very unlikely” in any of the three scenarios. This conservative threshold is consistent with Pierce et al.‘s (1996) original framework, which defined susceptibility as “the absence of a firm decision not to” engage in a behavior. This conservative threshold is particularly appropriate for wild mushroom consumption given the severe, often irreversible consequences of even a single consumption event—unlike tobacco use where health effects are cumulative, a single episode of consuming toxic mushrooms can result in acute organ failure or death. Therefore, identifying individuals who have not firmly rejected the possibility of consumption (i.e., those who did not select “very unlikely” for all scenarios) is epidemiologically meaningful for prevention targeting. This approach prioritizes sensitivity over specificity, which is appropriate when the consequences of missing at-risk individuals are severe.

**Algorithm Selection:** Three algorithms were selected based on their complementary strengths: Logistic Regression (interpretability and baseline performance), Random Forest (ensemble robustness), and Extremely Randomized Trees (hereafter ExtraTrees) (reduced overfitting through increased randomization).

**Machine Learning Modeling:** The analysis pipeline consisted of the following steps: (1) Data preprocessing: Missing values were handled using median imputation. (2) Feature scaling: StandardScaler was applied after train-test split to prevent data leakage. (3) Train-test split: The dataset was split into training (70%) and testing (30%) sets using stratified sampling to maintain outcome distribution balance. (4) Cross-validation: Five-fold cross-validation was conducted within the training set only to prevent information leakage from test data. (5) Hyperparameters: Default hyperparameters were used for all models (Logistic Regression: L2 penalty, C = 1.0; Random Forest and ExtraTrees: n_estimators = 100, max_depth = None). Extensive hyperparameter tuning was not pursued given sample size constraints and the associated risk of overfitting. (6) Model evaluation: Model performance was evaluated using accuracy and Area Under the Receiver Operating Characteristic Curve (AUC). (7) Confidence intervals: 95% confidence intervals for AUC were calculated via bootstrap resampling (1,000 iterations). (8) Feature importance: Feature importance was assessed through permutation importance analysis (100 permutations per feature).

All analyses were conducted using Python 3.11 with scikit-learn, pandas, and matplotlib libraries. Statistical significance was set at p < 0.05.

### 2.4. Ethics approval

This study was approved by the Research Ethics Committee of Royal Roads University (Protocol Li: 74/2024). This international collaboration involved the fourth author (Z.L.) who is affiliated with Royal Roads University, which served as the primary institutional sponsor. The approved protocol covered the full research design including data collection conducted at Fujian Polytechnic Normal University in China. All participants were informed about study purposes, procedures, and rights before completing questionnaires and provided written informed consent.

## 3. Results

### 3.1. Sample characteristics

Table 1 presents demographic and behavioral characteristics of 216 participants. The sample comprised 136 females (62.96%) and 80 males (37.04%), with second-year students representing the largest group (71 participants, 32.87%). The majority of participants (166, 76.85%) were from Fujian Province.

**Table 1:** Sample Characteristics (n = 216).

Characteristic	Category	n (%)
Gender	Male	80 (37.04)
	Female	136 (62.96)
Academic Year	First year	58 (26.85)
	Second year	71 (32.87)
	Third year	20 (9.26)
	Fourth year	67 (31.02)
Geographic Origin	Fujian Province	166 (76.85)
	Other provinces	50 (23.15)
Wild Mushroom Experience	Consumption experience	57 (26.39)
	Picking experience	50 (23.15)
	No experience	109 (50.46)

### 3.2. Media usage and trust patterns

Analysis of media usage patterns revealed that short video platforms (84.3%) and social media (83.8%) were primary information channels for university students. AI models emerged as the fifth most used platform, with 29.2% of participants reporting usage for information seeking (Table 2).

**Table 2:** Media Trust and Information Exposure Patterns (n = 216).

Category	Item	n (%)	Ranking
Media Trust	Newspapers/Magazines	173 (80.09)	1
	AI Models	164 (75.93)	2
	Television	161 (74.54)	3
	Radio	160 (74.07)	4
	Social Media	146 (67.59)	5
	Information Websites	145 (67.13)	6
	Short Video Platforms	140 (64.81)	7
	International Social Media	98 (45.37)	8
Information Exposure	Exposure to hallucinogenic content	185 (85.7)	–
	Frequent exposure to hallucinogenic content	47 (21.8)	–
	Perceive hallucinogenic content as dangerous	134 (62.0)	–
	Find hallucinogenic content interesting	58 (26.9)	–
	Exposure to prevention education	168 (77.78)	–

Note: Trust levels represent participants reporting “fairly trust and above” for each media type. Rankings apply only to media trust evaluation.

Trust evaluations were calculated only among users of each platform. For AI models, 63 participants (29.2% of the total sample) reported usage; among these users, 75.93% indicated trust levels of “fairly trust” or above, ranking second among the eight media types when comparing user-reported trust.

### 3.3. Information exposure and content attitudes

The majority of participants (185, 85.7%) had been exposed to wild mushroom hallucinogenic content on social media, with 21.8% reporting frequent exposure. While 62.0% perceived such content as dangerous, 26.9% found it interesting.

### 3.4. Sensitivity analysis

To assess the robustness of our findings, we conducted sensitivity analyses using three alternative susceptibility thresholds: (a) Stricter threshold (“Likely” or “Very likely” responses only): This resulted in a susceptibility rate of 40.7%, compared to 65.3% with the primary threshold. (b) Multiple scenario threshold (non-”Very unlikely” in at least 2 scenarios): This yielded a 52.3% susceptibility rate. (c) Continuous susceptibility score analysis: Mean susceptibility score was 1.67 (SD = 0.71) on the 1–4 scale. Key predictors (risk perception, mushroom picking experience) remained significant across all threshold definitions, supporting the robustness of our primary findings.

### 3.5. Susceptibility assessment

Table 3 presents behavioral intentions across three scenarios. Following the established susceptibility framework [8], participants were classified as susceptible if they indicated anything other than “very unlikely” in any scenario. Using this criterion, 141 participants (65.3%) were classified as susceptible to wild mushroom consumption.

**Table 3:** Behavioral Intentions Across Three Scenarios (n = 216).

Scenario	Very Unlikely (%)	Unlikely (%)	Likely (%)	Very Likely (%)	Likely + Very Likely (%)
Recent consumption intention	52.78	36.11	10.65	0.46	11.11
Future consumption intention	47.69	36.57	13.89	1.85	15.74
Friend invitation scenario	45.37	30.09	21.30	3.24	24.54

The temporal gradient observed across scenarios demonstrates that the friend invitation scenario showed the highest consumption susceptibility (24.54%), followed by future consumption within one year (17.59%), and recent consumption (11.11%).

### 3.6. Machine learning model performance

Among the three algorithms tested, Logistic Regression achieved the highest performance (AUC = 0.776, 95% CI: 0.679–0.862), followed by ExtraTrees (AUC = 0.745, 95% CI: 0.643–0.837) and Random Forest (AUC = 0.738, 95% CI: 0.634–0.831).

**Fig 1:**
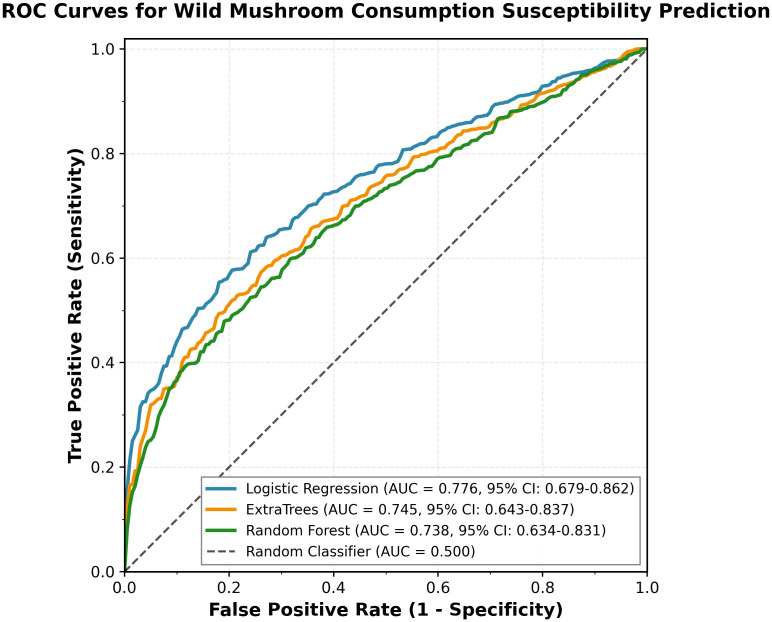
ROC curves for wild mushroom consumption susceptibility prediction.

Fig 1 presents the ROC curves for all three models. All models performed substantially better than random classification (AUC = 0.500). Cross-validation within the training set yielded mean AUC of 0.71 ± 0.05.

### 3.7. Feature importance analysis

Permutation importance analysis revealed the relative predictive contribution of each predictor variable to model performance. The importance values represent the mean decrease in model AUC when each feature is randomly shuffled, with higher values indicating greater predictive importance.

**Fig 2:**
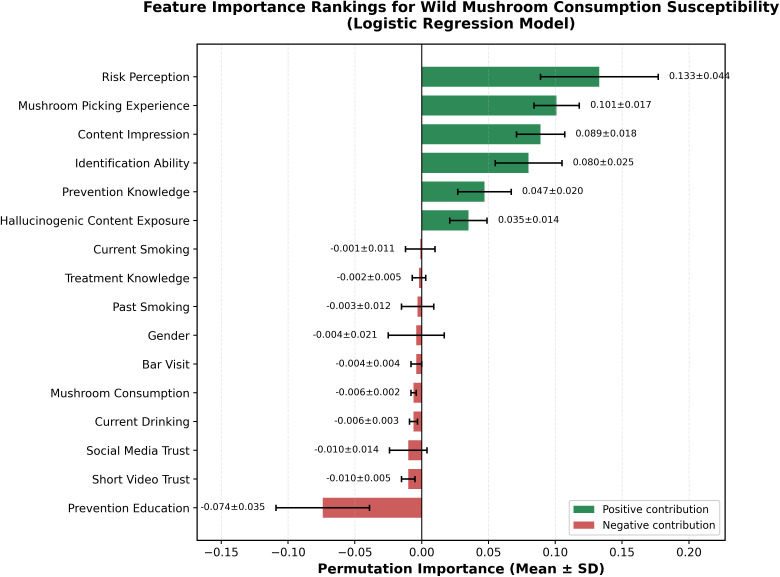
Feature importance rankings for wild mushroom consumption susceptibility.

Fig 2 presents feature importance rankings. Risk perception (0.133 ± 0.044) emerged as the strongest predictor, followed by mushroom picking experience (0.101 ± 0.017), content impression (0.089 ± 0.018), and identification ability (0.080 ± 0.025). Media usage and demographic factors showed relatively low importance values. These rankings were consistent across the three algorithms tested.

## 4. Discussion

### 4.1. Study limitations

Several important limitations must be acknowledged before interpreting the findings. First, the convenience sample of 216 students from a single university in Fujian Province limits generalizability across demographics, geographic regions, and cultural contexts. The sample is not representative of populations at highest risk for mushroom poisoning. Second, the cross-sectional design precludes causal inference; all relationships identified are associative rather than causal. Third, the sample size (12.7:1 ratio for variables), while adequate for exploratory analysis, limits the complexity of models that can be reliably estimated. Fourth, the trust measurement approach combines usage and trust within a single scale, making interpretation of non-user responses ambiguous. Fifth, self-reported measures may be subject to social desirability bias. Sixth, the AI trust finding (75.93%) represents trust among the minority of participants who use AI models (n = 63), not the full sample; the relatively small subsample limits the precision of this estimate and precludes generalization to non-users. These limitations should be considered when interpreting the findings discussed below.

### 4.2. Principal findings and cognitive factors

In this specific sample of Chinese university students, this exploratory study provides preliminary insights into factors associated with wild mushroom consumption susceptibility. The prominence of cognitive factors in prediction models is consistent with health behavior theory applications, particularly the Health Belief Model and Protection Motivation Theory, in understanding risk-related decision-making processes [9,15].

The superior performance of Logistic Regression over ensemble methods has important methodological implications. Cognitive predictors such as risk perception and identification ability are measured on ordinal Likert scales that inherently produce monotonic relationships with behavioral outcomes—higher perceived risk consistently associates with lower susceptibility. This monotonic structure is optimally captured by linear models, whereas Random Forest and ExtraTrees are designed to detect complex nonlinear interactions that may not exist in this predictor space. Consequently, the machine learning approach in this study did not uncover hidden nonlinear patterns; rather, it confirmed that straightforward linear relationships adequately characterize the predictor-outcome associations in this sample. The methodological contribution should therefore be understood as the systematic comparison of algorithms rather than the discovery of novel predictive patterns. Traditional regression approaches may be equally appropriate for similar health behavior prediction tasks involving cognitive predictors measured on ordinal scales.

Risk perception’s emergence as the strongest predictor (importance: 0.133 ± 0.044) is consistent with extensive literature demonstrating risk perception’s association with health behavior [8]. These associative findings from our cross-sectional design suggest that perceived risk may be relevant to consumption susceptibility in this population, though no conclusions can be drawn about causal direction, pending replication in diverse populations.

The predictive value of identification ability (0.080 ± 0.025) suggests that perceived knowledge may be associated with susceptibility. Individuals who believe they can identify poisonous mushrooms showed different susceptibility patterns, though whether this reflects actual knowledge or overconfidence cannot be determined from this study.

### 4.3. Susceptibility framework application

Within the constraints of this exploratory, single-site study, the application of the susceptibility framework to wild mushroom consumption behavior suggests potential utility beyond tobacco research. The 65.3% susceptibility rate observed in this study is comparable to susceptibility rates reported in other health behavior domains, though direct comparisons should be made cautiously given differences in populations and contexts [8]. These preliminary observations require validation across different cultural and demographic contexts.

The temporal gradient observed across scenarios (recent < future < friend invitation) is consistent with the original susceptibility framework, which emphasizes the importance of intentions and expectations for future behavior. This pattern demonstrates that susceptibility captures not only immediate behavioral intentions but also the cognitive flexibility that may lead to future risk behavior under different circumstances.

### 4.4. Social influence and media trust patterns

In this homogeneous sample of university students, the elevated consumption susceptibility in friend invitation scenarios (24.54% vs. 11.11% for recent consumption) is consistent with social influence effects documented in other health behavior research. This finding is consistent with Social Cognitive Theory’s emphasis on social modeling and peer influence [16]. Given the cross-sectional design, the direction of this association cannot be determined.

Regarding AI model usage, we observed that among the 29.2% of participants who reported using AI models for information seeking, trust levels were relatively high (75.93% reporting “fairly trust” or above). This descriptive finding suggests that AI platforms may be worth investigating as potential health communication channels among educated populations. However, several important caveats apply: trust in a platform does not indicate information accuracy; the effectiveness of AI-delivered health interventions remains untested; and substantial ethical considerations including misinformation risks and verification challenges would need to be addressed before any health communication applications. These descriptive findings require prospective validation before informing practice or policy.

### 4.5. Cultural considerations

Cross-cultural applicability requires careful consideration as the patterns observed in this Chinese university student sample may not generalize to other populations. Social influence mechanisms may vary across cultures with different collectivism-individualism orientations. Replication across diverse populations, age groups, and cultural contexts would be needed before broader conclusions can be drawn.

### 4.6. Implications for prevention strategies

The study’s findings suggest several potential directions for prevention strategies targeting Chinese university students, though these should be considered preliminary given the study’s limitations. The association between risk perception and susceptibility suggests that interventions enhancing accurate risk awareness may be relevant for this population. The observed influence of social contexts indicates that peer-based approaches might also merit consideration.

Future research should test intervention effectiveness through randomized controlled trials before drawing conclusions about effective prevention approaches.

## 5. Conclusions

This exploratory study demonstrates that cognitive factors, particularly risk perception and identification ability, show the strongest associations with wild mushroom consumption susceptibility among Chinese university students. These findings may inform the development of targeted prevention strategies for this demographic group, though replication is needed.

The exploratory application of the susceptibility framework to wild mushroom consumption behavior suggests potential utility of this framework beyond tobacco research. The 65.3% susceptibility rate observed highlights the substantial proportion of university students who may be cognitively predisposed to wild mushroom consumption under certain circumstances.

The observed trust levels in AI models among users represent a preliminary finding that may warrant further investigation in health communication research.

Important limitations of this study include the convenience sample from a single institution, cross-sectional design precluding causal inference, and the specific population studied. These findings should be considered hypothesis-generating rather than confirmatory.

Future studies should employ longitudinal designs to examine temporal relationships, expand to diverse populations including those at highest risk for mushroom poisoning, and test intervention effectiveness through randomized controlled trials.

## Supporting information

S1 File Survey instrument. Complete questionnaire used for data collection, including all demographic, media usage, cognitive assessment, and susceptibility measurement items. English version translated from original Simplified Chinese.
(DOCX)

S2 File Variable codebook. Detailed variable definitions including variable names, original Chinese text, English descriptions, response scales, coding schemes, and valid ranges for all study variables.
(XLSX)

S3 File Synthetic dataset. Synthetic dataset generated to mirror key statistical properties of the original data for reproducibility verification.
(XLSX)

S4 File Analysis code. Complete Python analysis code including data preprocessing, machine learning model training and evaluation, cross-validation procedures, bootstrap confidence interval calculation, and figure generation scripts.
(PY)
