# Supplementary material for: Wild mushroom consumption susceptibility among Chinese university students: A machine learning study
Source: PLoS One. 2026 Mar 24;21(3):e0345659. doi: 10.1371/journal.pone.0345659 (PMC13012498; doi:10.1371/journal.pone.0345659)
Supplement: S1 File — Complete questionnaire used for data collection, including all demographic, media usage, cognitive assessment, and susceptibility measurement items. English version translated from original Simplified Chinese. (DOCX) [file pone.0345659.s001.docx]

# S1 Questionnaire: Survey Instrument (English Version)

## Part I: Basic Information

**Q1. Gender:** [Single Choice] * - Male - Female

**Q2. Please select your ethnicity** [Single Choice] *

**Q3. Please enter your date of birth:** [Fill-in-the-blank] * _________________________________

**Q4. Grade/Academic Year:** [Single Choice] * - First Year - Second Year - Third Year - Fourth Year

**Q5. Geographic Origin** [Single Choice] * - Within Fujian Province _________________ - Outside Province _________________

**Q6. Do you drink alcohol?** [Single Choice] * - Never drink - Once a month or less - 2-4 times per month - 2-3 times per week - At least 4 times per week

**Q7. Do you smoke?** [Single Choice] * - Yes - No

**Q8. Have you ever smoked, even once?** [Single Choice] * - Yes - No

**Q9. In the past three months, have you been to bars (including lounges, nightclubs, etc.)?** [Single Choice] * - Yes - No

**Q10. Have you or your family members ever consumed wild mushrooms?** [Single Choice] * - Yes - No

**Q11. Have you or your family members ever picked wild mushrooms?** [Single Choice] * - Yes - No

## Part II: Media Use and Health Information Exposure

**A. What are the three main media sources you use to obtain information?** [Multiple Choice] * - [ ] Newspapers and magazines - [ ] Television - [ ] Radio - [ ] Information websites - [ ] Domestic social media (WeChat, Weibo, QQ, etc.) - [ ] Short video and other social media platforms (TikTok, Kuaishou, Bilibili, etc.) - [ ] AI large language models (such as Kimi, Doubao, Wenxin Yiyan, ChatGPT, etc.) - [ ] International social media (X/Twitter, Meta/Facebook, YouTube, Instagram, etc.) - [ ] Other _________________

**B. How much do you trust information from the following media sources?** [Matrix Single Choice] *

| **Media Source** | **Never Used** | **Completely Distrust** | **Distrust** | **Somewhat Distrust** | **Somewhat Trust** | **Trust** | **Completely Trust** |
| --- | --- | --- | --- | --- | --- | --- | --- |
| Newspapers and magazines | ○ | ○ | ○ | ○ | ○ | ○ | ○ |
| Television | ○ | ○ | ○ | ○ | ○ | ○ | ○ |
| Radio | ○ | ○ | ○ | ○ | ○ | ○ | ○ |
| Information websites | ○ | ○ | ○ | ○ | ○ | ○ | ○ |
| Domestic social media (WeChat, Weibo, QQ, etc.) | ○ | ○ | ○ | ○ | ○ | ○ | ○ |
| Short video and other social media platforms (TikTok, Kuaishou, Bilibili, etc.) | ○ | ○ | ○ | ○ | ○ | ○ | ○ |
| AI large language models (Kimi, Doubao, Wenxin Yiyan, ChatGPT, etc.) | ○ | ○ | ○ | ○ | ○ | ○ | ○ |
| International social media (X/Twitter, Meta/Facebook, YouTube, Instagram, etc.) | ○ | ○ | ○ | ○ | ○ | ○ | ○ |

## C. Wild Mushroom-Related Information Exposure and Cognition

**1. Have you seen content about wild mushroom hallucination (such as eating “Jian Shou Qing” and seeing “little people”) on social media, including videos and images/text?** [Single Choice] * - Never seen - Occasionally seen - Frequently seen

**2. What impression do these hallucinogenic videos leave on you?** [Single Choice] * - Find them interesting - Find them dangerous - Indifferent - Other _________________

**3. How likely are you to eat hallucinogenic wild mushrooms (such as “Jian Shou Qing”) recently?** [Single Choice] * - Very unlikely - Unlikely - Likely - Very likely

**4. In the next year, do you think you will eat hallucinogenic wild mushrooms (such as “Jian Shou Qing”)?** [Single Choice] * - Very unlikely - Unlikely - Likely - Very likely

**5. In the next year, if your best friend invites you to eat hallucinogenic wild mushrooms (such as “Jian Shou Qing”), would you eat them?** [Single Choice] * - Very unlikely - Unlikely - Likely - Very likely

**6. Please indicate your knowledge about wild mushrooms (1=Strongly Disagree, 5=Strongly Agree)** [Matrix Scale] *

| **Statement** | **Strongly Disagree** | **Disagree** | **Neutral** | **Agree** | **Strongly Agree** |
| --- | --- | --- | --- | --- | --- |
| I can distinguish between poisonous and edible wild mushrooms | ○ | ○ | ○ | ○ | ○ |
| I know the types of poisonous wild mushrooms | ○ | ○ | ○ | ○ | ○ |
| I can identify typical characteristics of poisonous mushrooms | ○ | ○ | ○ | ○ | ○ |
| High-temperature cooking can completely remove toxins from wild mushrooms | ○ | ○ | ○ | ○ | ○ |
| Wild mixed mushrooms can be stored and cooked together | ○ | ○ | ○ | ○ | ○ |
| Wild mushroom poisoning can cause symptoms such as nausea and vomiting | ○ | ○ | ○ | ○ | ○ |
| Not drinking alcohol when eating wild mushrooms can prevent some types of wild mushroom poisoning | ○ | ○ | ○ | ○ | ○ |
| There is no specific antidote for wild mushroom poisoning | ○ | ○ | ○ | ○ | ○ |
| Wild mushroom poisoning can lead to death in severe cases | ○ | ○ | ○ | ○ | ○ |
| One should immediately call 120 (emergency services) after wild mushroom poisoning | ○ | ○ | ○ | ○ | ○ |
| After wild mushroom poisoning, simple self-induced vomiting should be performed | ○ | ○ | ○ | ○ | ○ |
| Not picking or eating wild mushrooms is the best method to prevent wild mushroom poisoning | ○ | ○ | ○ | ○ | ○ |
| Hallucinogenic wild mushroom poisoning may cause permanent damage including nervous system damage | ○ | ○ | ○ | ○ | ○ |

**7. Have you watched educational videos or seen promotional materials about wild mushroom poisoning prevention?** [Single Choice] * - Never seen - Have seen

## Part III: Protection Motivation Assessment

(1=Strongly Disagree, 5=Strongly Agree)

### A. Threat Appraisal

**1. Perceived Severity** [Matrix Scale] *

| **Statement** | **Strongly Disagree** | **Disagree** | **Neutral** | **Agree** | **Strongly Agree** |
| --- | --- | --- | --- | --- | --- |
| Wild mushroom poisoning seriously threatens life safety | ○ | ○ | ○ | ○ | ○ |
| Treatment costs for wild mushroom poisoning create a heavy financial burden for families | ○ | ○ | ○ | ○ | ○ |
| Wild mushroom poisoning affects study and daily life | ○ | ○ | ○ | ○ | ○ |

**2. Perceived Susceptibility** [Matrix Scale] *

| **Statement** | **Strongly Disagree** | **Disagree** | **Neutral** | **Agree** | **Strongly Agree** |
| --- | --- | --- | --- | --- | --- |
| Anyone eating wild mushrooms may encounter poisoning risks | ○ | ○ | ○ | ○ | ○ |
| Even experienced people may mistakenly eat poisonous mushrooms | ○ | ○ | ○ | ○ | ○ |
| It is difficult to distinguish whether wild mushrooms are poisonous in restaurants | ○ | ○ | ○ | ○ | ○ |

**3. Perceived Benefits** [Matrix Scale] *

| **Statement** | **Strongly Disagree** | **Disagree** | **Neutral** | **Agree** | **Strongly Agree** |
| --- | --- | --- | --- | --- | --- |
| Wild mushrooms are rare delicacies | ○ | ○ | ○ | ○ | ○ |
| Eating wild mushrooms allows one to experience local specialties | ○ | ○ | ○ | ○ | ○ |
| Sharing wild mushrooms with family and friends enhances relationships | ○ | ○ | ○ | ○ | ○ |

**4. Perceived Costs** [Matrix Scale] *

| **Statement** | **Strongly Disagree** | **Disagree** | **Neutral** | **Agree** | **Strongly Agree** |
| --- | --- | --- | --- | --- | --- |
| Purchasing wild mushrooms increases economic burden | ○ | ○ | ○ | ○ | ○ |
| Finding wild mushrooms consumes considerable time | ○ | ○ | ○ | ○ | ○ |
| Eating wild mushrooms may bring health risks | ○ | ○ | ○ | ○ | ○ |

### B. Coping Appraisal

**1. Response Efficacy** [Matrix Scale] *

| **Statement** | **Strongly Disagree** | **Disagree** | **Neutral** | **Agree** | **Strongly Agree** |
| --- | --- | --- | --- | --- | --- |
| Completely avoiding wild mushrooms is the safest approach | ○ | ○ | ○ | ○ | ○ |
| Learning to identify poisonous mushrooms is very necessary | ○ | ○ | ○ | ○ | ○ |
| Improving safety awareness can effectively prevent poisoning | ○ | ○ | ○ | ○ | ○ |

**2. Self-Efficacy** [Matrix Scale] *

| **Statement** | **Strongly Disagree** | **Disagree** | **Neutral** | **Agree** | **Strongly Agree** |
| --- | --- | --- | --- | --- | --- |
| I am confident in refusing any form of wild mushrooms | ○ | ○ | ○ | ○ | ○ |
| I can explain to family and friends the reasons for not eating wild mushrooms | ○ | ○ | ○ | ○ | ○ |
| I can persuade people around me to take wild mushroom safety seriously | ○ | ○ | ○ | ○ | ○ |

**3. Response Costs** [Matrix Scale] *

| **Statement** | **Strongly Disagree** | **Disagree** | **Neutral** | **Agree** | **Strongly Agree** |
| --- | --- | --- | --- | --- | --- |
| Refusing wild mushrooms might make family and friends unhappy | ○ | ○ | ○ | ○ | ○ |
| Not eating wild mushrooms means missing some culinary experiences | ○ | ○ | ○ | ○ | ○ |
| Refusing family and friends’ wild mushroom recommendations feels embarrassing | ○ | ○ | ○ | ○ | ○ |

## Administration Notes

**Original Language:** Simplified Chinese (中文简体)

**Administration Mode:** Online survey via Wenjuanxing platform

**Target Population:** University students in Fujian Province, China

**Data Collection Period:** August–September 2024

**Ethics Approval:** Royal Roads University Research Ethics Committee (Protocol Li: 74/2024)

**Estimated Completion Time:** 8–12 minutes

**Quality Control:** Questionnaires completed in less than 90 seconds were excluded from analysis.

## End of Questionnaire

Thank you for your participation.
